# Parental Insightfulness and Its Association With Social Competence in Autistic and Non‐Autistic Children

**DOI:** 10.1002/aur.70127

**Published:** 2025-10-15

**Authors:** Liron Oliver‐Aronson, Lital Kohn, Tali Gev, Ofer Golan

**Affiliations:** ^1^ Department of Psychology Bar‐Ilan University Ramat‐Gan Israel; ^2^ OTI ‐ The Israeli Autism Association Givat‐Shmuel Israel

**Keywords:** autism, parental insightfulness, reflective functioning, social competence, social skills

## Abstract

Parental insightfulness (PI), the parent's capacity to reflect upon their own and their child's mental and emotional states, has been associated with various aspects of children's socio‐emotional development. This study examined PI regarding child‐peer interactions and its association with social competence in autistic and non‐autistic (NA) children, aged 4–7 years. We hypothesized that parents of autistic and NA children would demonstrate different patterns of PI and that PI would moderate the association between autism diagnosis and social competence. Participants included 68 autistic children and their parents and 46 NA children and their parents. Parents watched videos of their child playing with a peer and completed the Insightfulness Assessment (IA) interview. They also reported on their child's social competence and their own parental reflective functioning. Results revealed that compared to NA children's parents, parents of autistic children showed similar levels of positive insightfulness about their child but had greater difficulties maintaining focus on their child's mental states, showed less acceptance, and more concern about the child. PI moderated the negative association between autism diagnosis and children's social competence so that in higher PI levels, the association was weaker than in lower PI levels. This study's findings suggest higher PI may mitigate social challenges for autistic children. Hence, PI and its nuances may be an intervention target for autistic children's parents with the aim of improving children's social outcomes.

**Summary**

- The current study examined the insightfulness of parents (the parent's understanding of the child's mental and emotional states) on their child's interaction with peers among parents to autistic and non‐autistic children, and its association with the child's social competence.
- Findings revealed that autistic children's parents maintained the same level of positive insightfulness as parents to non‐autistic children but showed lower levels of focus on their child and acceptance of the child's behavior, and more concern than parents to non‐autistic children.
- Parental insightfulness played a role in the social competence of autistic (compared to non‐autistic) children, such that a higher level of parental insightfulness was associated with better social competence of autistic children.

## 1 Introduction

Autism is a neurodevelopmental condition, characterized by altered social communication and restricted, repetitive behaviors and interests (American Psychiatric Association 2013). The prevalence of autism is currently reported at around 1.83% of 4–6‐year‐olds (Dinstein et al. 2024). A key challenge in autism lies in the development of social competence, that is, the ability to evaluate social situations, to recognize others' feelings and intentions, and to select context‐appropriate social behaviors (American Psychiatric Association 2023). Social competence in childhood has been consistently linked to positive developmental outcomes including peer acceptance and social adjustment (Choi and Juhu 2003; Sette et al. 2017), academic success in early school years (Cooper et al. 2014), and mental health and well‐being (Carter et al. 2004; Denham et al. 2015). Social competence in early childhood is also associated with positive outcomes in early adulthood across multiple domains of education, employment, and mental health (Jones et al. 2015; Nandy et al. 2020).

Social competence deficits in young autistic children are persistent and pervasive over and above cognitive and language abilities (Yavuz et al. 2017). Autistic children demonstrate impairments across the three core components of social competence. They experience difficulties in assessing social situations, initiating or maintaining interactions, and managing social conflicts (McNair et al. 2025). In addition, autistic children have significant challenges recognizing emotions and understanding the intentions of others based on nonverbal cues such as facial expressions, vocal intonation, or body language (Fridenson‐Hayo et al. 2016). Finally, studies demonstrate that autistic children exhibit difficulties adapting their behavior to different social contexts, and that they often display disregulated or socially unacceptable behaviors (Bar Yehuda and Bauminger‐Zviely 2024). Consequently, autistic children show difficulties forming, maintaining, and being reciprocal in friendships (Freeman et al. 2015). These difficulties lead to experiences of loneliness and peer rejection, which can contribute to mood and anxiety disorders (Bauminger and Kasari 2000; Rao et al. 2008). Given their social competence deficits, autistic children may need increased parental support and mediation in the social realm (Vasina 2020).

Parents serve as social competence models to their children from early development. They explain and mediate adaptive behaviors in various settings, thus establishing a framework for how children interpret their social world (Edwards et al. 2010; Lynch and Simpson 2010). Parents' ability to effectively support the development of their child's social competence requires taking the child's mental and emotional perspective, known as parental insightfulness (PI; Koren‐Karie and Oppenheim 2018; Oppenheim and Koren‐Karie 2002).

PI is defined as the parent's ability to take into consideration the motives that underlie the child's behavior while accepting the child as a separate, whole, and integrative individual with both positive and challenging aspects. An insightful parent views, without distortion or excessive criticism, both the expected and the unexpected aspects of the child's behavior (Oppenheim and Koren‐Karie 2014; Shahar‐Maharik et al. 2018). PI may be hampered by anger at the child or worries about him and his difficulties, which restrict the parent's flexibility in thinking about the child's behavior, experience, and motives. PI may also be compromised when the parent is distracted by other issues (including the parent's own experiences, thoughts, and feelings) rather than focused on the child (Oppenheim and Koren‐Karie 2014; Shahar‐Maharik et al. 2018).

The Insightfulness Assessment–(IA, Koren‐Karie et al. 2002) is a semi‐structured interview that examines the above‐mentioned aspects of PI in reference to the child's interaction with the parent. IA coding includes ten scales, grouped into two overarching dimensions. The first, Positive Insight, includes the scales *insight, complexity, openness, acceptance, richness, and coherence*. It reflects a parent's ability to describe the underlying motives behind their child's behavior in a coherent manner. The second, Focus on Child, includes the scales *maintaining focus on the child, separateness, hostility/anger*, and *concern*. It reflects the parent's capacity to remain focused on the child while recognizing that the child's motives are distinct from their own (Siller et al. 2018). Conceptually, these two dimensions align with a two‐dimensional model of parenting that emphasizes both sensitivity and autonomy‐supportive, non‐intrusive parenting, reflecting the core principles of insightfulness (Gomez et al. 2018).

PI has been shown to promote the child's own capacity for reflective functioning, which in turn is thought to foster self‐regulation and productive, intimate, and sustaining relationships with family and peers (Cooper and Redfern 2015; Ensink and Mayes 2010). Studies indicate the importance of PI in the development of the child's socio‐emotional competence (Koren‐Karie and Oppenheim 2018). Higher levels of insightfulness have been related to greater emotional availability of caregivers, and to their children exhibiting more competent social information processing and better social behavior in preschool settings (Ziv et al. 2018). Oppenheim and Koren‐Karie (2002) showed that mothers' insightfulness predicted later theory of mind of the child. Benbassat and Priel (2012) found that parents' ability to reflect upon their adolescent's mental and emotional states is correlated with the adolescent's own reflective functioning and social competence. Finally, maternal insightfulness and secure attachment during infancy have been found to predict later adolescent insightfulness towards a close friend (Shahar‐Maharik et al. 2018). On the other hand, impairment in the parent's ability to reflect on their child was related to hindered social and emotional development of the child (Nijssens et al. 2020).

Regarding children's interactions with peers, Mize et al. (1995) have shown that mothers' perception of their child as skilled in interpersonal interactions was related to reduced supervision of the child's peer‐play activities. Furthermore, children displaying lower levels of social competence tend to elicit higher levels of maternal supervisory engagement (Ladd and Pettit 2002). This maternal scaffolding emerged as a central predictor of later social competence among children with atypical development (Paczkowski and Baker 2007).

Parents who are insightful frame their children's motives and behaviors within a framework of acceptance with the active intention to find reasonable explanations for their child's behavior, especially when responding to challenging behaviors (Oppenheim and Koren‐Karie 2002, 2014). This is why PI may play an even more important role when children struggle to socially communicate their needs and mental and emotional states, and rely on their parents as social mediators, such as in the case of autism (Oppenheim and Koren‐Karie 2014). In their systematic review, Oppenheim and Koren‐Karie (2018) showed that PI of parents to autistic children is similar to PI of parents to NA children. Their review demonstrated no relationship between PI and severity of the child's diagnosis on the autism spectrum. It has been argued that insightful parents, who can reflect on their autistic children's minds, and comprehend their motivations and challenges, may offer them more effective social mediation (Oppenheim et al. 2012; Park et al. 2023). Studies have shown that PI derived behavior—being synchronous, responsive, and attuned to the child is associated with subsequent communication skills in autistic children such as joint attention and language development (Siller and Sigman 2002). Moreover, early PI predicted inclusive educational placement for autistic children at a 4–8‐year follow‐up (Dolev et al. 2014).

Parents, with their intimate knowledge of their autistic child's abilities, behaviors, and emotional states (Rogers et al. 2019; Siller et al. 2018), play a crucial role in mediating social interactions. Autistic children particularly rely on their parents in peer contexts, where their social difficulties may be most evident (Park et al. 2023). Hence, it is plausible that PI may enhance the quality of parental mediation, which in turn may augment the child's social competence.

However, since the verbal and the nonverbal communication of socio‐emotional signals is altered among autistic children (Begeer et al. 2008; Dolev et al. 2009; Mitchell et al. 2006), parents may need to put more effort into understanding their autistic child's internal experience (Conti 2015). Moreover, parents of autistic children often experience chronic stress and worry (Bravo‐Benítez et al. 2019; Schieve et al. 2007), which may affect their PI (Bateman and Fonagy 2004). Finally, parents' awareness of their child's autism may raise their concern about the child's social functioning (Azad and Mandell 2016; Dillenburger et al. 2010; Richards et al. 2016). Studies have demonstrated that parental stress during children's developmental periods is strongly influenced by their children's challenges with social interactions (DesChamps et al. 2020; Hayes and Watson 2013; Hutchison et al. 2016). Hence, it would be interesting to multidimensionally examine the various aspects of PI when autistic children's parents reflect on the child's peer interaction.

### 1.1 The Current Study

So far, little is known about the nature of the interactions between autism, PI, and social competence. Therefore, examining a parent's ability to understand their child's motivations, behavior, and emotion in the context of peer interactions can extend and elaborate insights into the relationship between PI and autistic children's social competence.

Previous studies comparing PI of parents to autistic and NA children have not found significant group differences. However, these studies examined PI dichotomously (i.e., insightful or not) and did not delve into the various components of PI which may differ between parents of autistic and NA children (e.g., Oppenheim and Koren‐Karie 2018). In addition, previous studies examined how parents reflect on their children's minds in the context of parent–child interaction. The current study offers a unique view of parents' reflection on their child's emotions, motives, and behavior while they are watching their child interacting with a peer. For that aim, we adapted the IA (Koren‐Karie et al. 2002) so that it measures PI when the child interacts with a peer, rather than with the parent.

To examine PI more comprehensively, it was measured both through scored interviews (in a peer‐interaction context) and via parent self‐report (in a parent–child relationship context). Looking at group differences on PI components, we hypothesized that parents of autistic and NA children will demonstrate different patterns of PI when reflecting on their child during peer interaction, but similar patterns of PI when reflecting upon their parent–child relationship.

Since autistic children benefit from parental social mediation in social interactions and may depend on it even more than their NA peers (Park et al. 2023), and since research has demonstrated the relationship between PI and children's emotional and social competencies (Cooper and Redfern 2015), we hypothesized that the association between group (Autism/NA) and the child's social competence will be moderated by PI, so that social competence differences between autistic and NA children will get smaller the higher the level of their parents' insightfulness.

## 2 Methods

### 2.1 Participants

One hundred and fourteen participants were recruited to the study. The autism group comprised 68 autistic children aged 4–7 years (*M* = 5.33, SD = *0*.95; 60 males, 8 females) with a pre‐existing diagnosis of ASD without intellectual or language impairment, and their parents aged 28–51 years (*M* = 40.36, SD = 4.5; 59 mothers, 9 fathers). They were recruited through the *Bayit‐Echad* autism clinical centers of OTI—the Israeli Autism Association, as part of a large‐scale clinical trial. Participants' diagnosis was confirmed using the Autism Diagnostic Observation Scale, 2nd edition (ADOS; Lord et al. 2012).

The NA group comprised 46 children aged 4–7 years (*M* = 5.29, SD = 1.12; 31 males, 15 females), with no reported neurodevelopmental conditions, and with no sibling diagnosed with ASD, and their parents aged 25–47 years (*M* = 36.43, SD = 5.65; 48 mothers, 3 fathers). The families in the NA group were recruited for a child social interaction study through social media. Intellectual functioning of both groups was assessed using two subscales from the fourth edition of the Wechsler Intellectual Scale for Children (WPPSI‐III‐Heb; Wechsler 2012): Vocabulary and matrix reasoning. Participants' performance on the two subscales did not fall below two standard deviations of the mean. Groups were comparable on age, cognitive abilities, family status, and family income, but differed on sex, which was therefore controlled in all analyses. The groups' background characteristics are summarized in Table 1.

**TABLE 1:** Participants' demographic data.

Background variable	NA (*n* = 46)		Autism (*n* = 68)		*t*(112)
	Mean (SD)	Range	Mean (SD)	Range	
Child age (years)	5.29 (1.12)	3.83–7.25	5.33 (0.95)	3.83–7.42	0.19
WPPSI‐III vocabulary	10.70 (2.44)	7.00–17.00	9.97 (2.93)	6.00–19.00	−1.38
WPPSI‐III matrix reasoning	11.02 (3.12)	4.00–15.00	11.40 (2.95)	5.00–19.00	0.65
Family income^a^	51.87		61.31		*Z* = −1.58
Child sex (m:f)	31:15		60:8		*Χ* ^***2***^(1) = 7.41**

Abbreviations: NA, non‐autistic; WPPSI‐III—Wechsler Preschool and Primary Scale of Intelligence, 3rd Ed.

^a^ Mann–Whitney test. Mean ranks are provided; ** *p* < 0.01. *p* > 0.1 for all other comparisons.

### 2.2 Measures

#### 2.2.1 Child Measures (Social Competence)

***Vineland Adaptive Behavior Scales‐Third Edition (VABS‐3)*** (Sparrow et al. 2016). The VABS‐3 is a structured parent interview designed to assess adaptive behavior from infancy to adulthood. It consists of 11 subscales, which are clustered into four domains: communication, daily living skills, socialization (e.g., “Asks others if they want to hang out or play with him”), and motor. For the current study, only the socialization scale was used. Responses are scored on a Likert scale (0 = “Never”, 1 = “Sometimes”, 2 = “Usually”), reflecting the level of support the child needs in performing each behavior. The VABS‐3 yields standard domain scores, with *M* = 100 and SD = 15. Higher scores indicate more adaptive behavior. The VABS‐3 demonstrates high internal consistency on all domains (0.86–0.97; Pepperdine and McCrimmon 2018).

***Social Responsiveness Scale‐Second Edition (SRS‐2)*** (Constantino and Gruber 2012). The SRS‐2 is a 65‐item rating scale designed to measure the level of autism spectrum symptoms as they manifest in natural social settings. It consists of five subscales: social awareness, social cognition, social communication, social motivation, and restricted repetitive behaviors subscales. Items are scored on a 4‐point Likert scale, ranging from 1 (“Not true”) to 4 (“Almost always true”). The SRS‐2 yields T‐scores for each subscale (*M* = 50, SD = 10) as well as a social communication index (SCI), which comprises all the social subscales. For the current study, only the SCI was used. Higher T‐scores indicate poorer social competence. The internal consistency for the SRS‐2 parent report in Israel was reported as *α* = 0.94 (Rabin et al. 2018).

***Social Skills Improvement System (SSIS)*** (Gresham and Elliott 2008). The SSIS is a 76‐item standardized rating scale which assesses global social competence and behavior problems in children aged 3–18 years. It consists of two standard composite scales: social skills (SS) and behavior problems (BP). In this study, only the social skills domain was used. Items are scored on a 4‐point Likert scale, ranging from 0 (“Never”) to 3 (“Almost always”). The SSIS yields standard scores for each scale (*M* = 100, SD = 15). A higher score on the social skills domain indicates a higher level of social competence. Internal consistency for the SSIS in Israel was *α* = 0.85 (Rabin et al. 2018).

#### 2.2.2 Parent Measures (Parental Insightfulness)

***Insightfulness Assessment (IA)*** (Koren‐Karie et al. 2002). The IA is a semi‐structured interview designed to evaluate parents' capacity for reflection and evaluation of their child's emotional and mental states, based on video segments of the child that are played to the parent. In the current study, each parent watched three video segments of their child engaged in ecologically valid examiner‐guided interactions with a peer. The activities were: (1) A *competitive* game (*Simon says*), in which each child took part both as the leader and as the follower; (2) a *cooperative* game, in which children were asked to cooperate on a *Jenga* game, trying together to prevent the brick tower from collapsing; (3) an *unstructured* drawing activity. Parents were shown 2‐min segments from each interaction. For the Simon Says game, parents watched a segment showing the role transition between the participant and the peer, allowing them to observe their child in both leader and follower roles. As for the Jenga game, parents watched 90 s before and 30 s after the collapse of the tower. Lastly, during the drawing activity, a two‐minute segment was taken, starting 30 s after the researcher introduced the activity. After watching each segment, parents were asked to discuss their impressions of the child's thoughts and feelings, as well as the parent's own thoughts and feelings, according to the IA protocol (see Appendix S1).

The interview was recorded and transcribed verbatim. IA transcripts were scored by a trained and reliable rater, who was naïve to the children's diagnosis and to the research hypotheses. Inter‐rater reliability with the IA's creator was assessed for 20% of the interviews. Reliability for the 10 IA scales ranged between Kappa = 0.80–0.95 for all, except for the Maintenance of Focus, which was Kappa = 0.57. Scoring was conducted on ten 9‐point rating scales. Scales included: Insight into the Child's Motives; Openness/Flexibility of Thought; Complexity and Richness in Description of the Child; Maintenance of Focus on Child; Acceptance/Warmth; Hostility/Anger; Concern regarding the child; Richness of Description of Child; Separateness from Child; and Coherence of Thought (see Appendix S2). In addition, the validity and the use of overarching factors of IA scores for the current peer‐interaction‐focused interview were examined.

***Parental Reflective Functioning Questionnaire (PRFQ)*** (Luyten et al. 2017). The PRFQ was chosen due to the theoretical resemblance between Parental Insightfulness and Parental Reflective Functioning (Koren‐Karie and Oppenheim 2018). The PRFQ is an 18‐item rating scale providing a brief, multidimensional self‐report of parental reflective functioning. It includes three domains: (1) parental interest and curiosity in their child's mental states (IC, e.g., “I wonder a lot about what my child is thinking and feeling”), (2) parental certainty of their child's mental states (CMS, e.g., “I always know what my child wants”), and (3) parental pre‐mentalizing modes (PM, e.g., “My child cries around strangers to embarrass me”).

Items on the PRFQ are rated on a 7‐point Likert scale, ranging from 1 (“Strongly disagree”) to 7 (“Strongly agree”). For the IC and CMS domains, higher scores represent higher levels of PI. The PM domain is reversed, that is, higher scores indicate a non‐mentalizing stance, which includes malevolent attributions and difficulties entering the child's subjective world. Internal consistency (Cronbach's *α*) for the PRFQ scales was reported as 0.70, 0.82, and 0.75 for PM, CMS, and IC, respectively (Luyten et al. 2017).

### 2.3 Procedure

Ethical approval for the study was obtained from Bar‐Ilan university IRB. Parents in the autism group gave their informed consent in writing, whereas parents in the NA group provided consent electronically through the Qualtrics platform.

Each family was paid two home visits. For the first meeting, parents were asked to schedule a playdate for their child with a peer the child has known for at least 1 month who is within a range of 1.5 years older or younger than their child. The peer's parents provided electronic consent for their child to be videotaped while playing with the participant.

Participants and their peers engaged in a 10‐min examiner‐guided peer interaction. The interaction included the three above‐mentioned activities and was videotaped for use in the IA interview. After the meeting, parents completed the SRS‐2, the SSIS, and the PRFQ through the Qualtrics platform. In the second meeting, the child underwent two intelligence sub‐tests (vocabulary and matrix reasoning), and parents were given the IA. After these two meetings, a trained research assistant, who was naive to the child's diagnosis, administered the VABS‐3 to the parent. Participants were monetarily compensated for their time and effort.

## 3 Results

First, missing data analysis was conducted to assess the extent and patterns of missingness within the dataset. The results indicated that 10 participants had not completed all the questionnaires, that is, approximately 2% of the data was missing across various variables. Data were missing at random, based on Little's MCAR test (*χ* ^2^ = 21.59, *p* = 0.2), indicating that the missingness was unrelated to observed or unobserved data. Subsequently, missing values were imputed using the Expectation–Maximization (EM) algorithm in SPSS (version 25).

Next, to confirm the groups differed on the three social competence measures (SRS‐SCI, SSIS‐SS, and VABS Socialization scale), a Multivariate Analysis of Covariance (MANCOVA) was conducted, with sex as a covariate. The overall analysis yielded a statistically significant effect of group (*F*_Wilks_(3,109) = 45.14, *p* < 0.001, *η^2^* = 0.56), indicating that the groups significantly differed in their overall social competence across the three measures. In addition, sex had an overall effect as a covariate (*F*_Wilks_(3,109) = 3.31, *p* < 0.05, *η^2^* = 0.08). Univariate analyses, detailed in Table 2 revealed that compared to the NA group, the autism group showed significant difficulties on the three social competence measures. In addition, sex had a significant effect as a covariate on the SSIS‐SS (*F*_Wilks_(1,111) = 6.37, *p* < 0.05, *η^2^* = 0.05) and the SRS‐SCI (*F*_Wilks_(1,111) = 5.88, *p* < 0.05, *η^2^* = 0.05).

**TABLE 2:** Group comparisons on the three social comptenece measures.

Measures	NA			Autism			*F*(1, 111)	*η^2^*
	*M*	SD	Range	*M*	SD	Range		
SRS‐SCI	49.04	6.01	38–64	64.79	9.74	45–88	105.55***	0.49
SSIS‐SS	94.07	9.82	72–125	86.72	11.95	59–117	16.50***	0.13
VABS socialization	101.11	6.8	85–117	87.96	8.65	70–112	69.37***	0.39

Abbreviations: NA, non‐autistic; SRS–SCI, Social Responsiveness Scale–Social Communication Index; SSIS–SS, Social Skills Improvement Scales–Social Skills; VABS, Vineland Adaptive Behavior Scales.

*** *p* < 0.001.

To examine if IA scoring, based on parents' reflections on their child's interaction with a peer, maintains the same factor structure as the original IA, focused on the child's interaction with a parent, a Principal Component analysis with varimax rotation was conducted for the entire sample on nine of the IA scales, with the exception of hostility/anger, which had no variance. As expected, two factors with Eigenvalues greater than 1.0 were extracted. The first factor, *Positive Insightfulness*, accounted for 63.8% of the variance and included the following scales (factor loadings in parentheses): complexity/Balance in description of child (0.95), Richness of description of child (0.97), Coherence of thought (0.97), Insight into child's motives (0.96), Acceptance/warmth (0.92), and Openness (0.92). The second factor, *Focus on Child*, accounted for 18.1% of the variance and included the following scales: Maintenance of focus on child (0.82), Separateness from child (0.65), and Concern regarding the child (−0.83). The factor structure was identical to that found in the original IA factor analysis (Gomez et al. 2018). Next, two aggregate scores were formed by averaging the scales within each factor. Scores of the factor analysis are detailed in Table 3.

**TABLE 3:** Factor analysis with varimax rotation factors of the IA scales (*n* = 114).

Item	Factor's loadings	
	Factor 1‐positive insight	Factor 2‐focus on child
Richness	0.97	
Coherence	0.97	
Insight	0.96	
Complexity	0.95	
Acceptance	0.92	
Flexibility	0.92	
Concern (reversed)		0.83
Focus on child		0.82
Separateness		0.66
Mean (SD)	5.66 (1.47)	8.17 (0.75)
Range	1–8.7	5.67–9
Eigenvalue	5.74	1.63
% of variance	63.8%	18.1%
Cronbach's alpha	0.98	0.67

Abbreviation: IA, insightfulness assessment.

Next, we examined group differences on the two PI measures: To test group differences on the semi‐structured PI interview (IA), two MANCOVA analyses were conducted, one on the two IA dimensions (Positive Insight and Focus on Child), and another on the nine IA scales (excluding hostility/anger, which had no variance). Group was the independent variable, and sex was a covariate in all analyses. The first MANCOVA yielded a marginally significant overall effect of group (*F*_Wilks_(2,110) = 3.02, *p* = 0.05, *η^2^* = 0.05). In addition, sex had an overall effect as a covariate (*F*_Wilks_(2,110) = 3.28, *p* < 0.05, *η^2^* = 0.06). Univariate analyses, detailed in Table 4 revealed significant group differences only on the Focus on Child dimension, signifying a lower degree of parental focus in parents of autistic children, compared to parents of NA children. Similarly, sex as a covariate was significant only for Focus on Child (*F*_Wilks_(1,111) = 6.61, *p* < 0.05, *η^2^* = 0.06).

**TABLE 4:** Means (SD) of the two IA dimensions in the Autism/NA groups.

IA dimensions	NA	Autism	*F*(1, 111)	*η^2^*
Positive insight	5.90 (1.29)	5.5 (1.7)	2.5	0.02
Focus on child	8.31 (0.65)	8.08 (0.8)	5.08*	0.04

Abbreviations: IA, insightfulness assessment; NA, non‐autistic.

* *p* < 0.05.

The second MANCOVA, examining the 9 IA scales, did not yield an overall group effect (*F*_Wilks_(9,103) = 1.25, *p* = 0.28, *η^2^* = 0.10). However, univariate analyses, detailed in Table 5 revealed significant group differences in three of the IA scales (Acceptance, Focus on child, and Concern). These results indicated that parents of autistic children demonstrated higher levels of concern and lower levels of acceptance and focus on their child's thoughts and feelings during the semi‐structured interview, compared to parents of NA children. Sex had no significant effect.

**TABLE 5:** Means (SD) of the 9 IA Scales in the Autism/NA groups.

Scales	NA	Autism	*F*(1, 111)	*η^2^*
Complexity	6.12 (1.3)	5.82 (1.58)	1.33	0.01
Separateness	8.14 (0.82)	8.1 (0.87)	0.55	0.00
Coherence	6.02 (1.39)	5.6 (1.8)	2.12	0.02
Openness\flexibility	5.23 (1.04)	4.96 (1.33)	1.85	0.02
Richness	5.91 (1.43)	5.5 (1.57)	2.21	0.02
Concern	1.89 (1.11)	2.21 (1.19)	4.12*	0.04
Focus on child	8.67 (0.56)	8.35 (0.99)	5.71*	0.05
Acceptance	6.22 (1.38)	5.62 (1.77)	4.78*	0.04
Insight	5.87 (1.6)	5.47 (1.75)	1.87	0.02

Abbreviation: IA, insightfulness assessment.

* *p* < 0.05.

To examine group differences on the three domains of the self‐reported PRFQ (IC, CMS, and PM), a MANCOVA was conducted, with sex as a covariate. No significant group differences were found for the overall effect (*F*_Wilks_(3,109) = 0.80, *p* = 0.49, *η^2^* = 0.02) or for the univariate analyses (see Appendix S3).

Next, we examined the moderating role of PI on the association between having autism and the child's social competence. To examine the moderating effect of PI as measured through the IA interview, six moderation analyses were conducted, predicting VABS Socialization, SSIS‐SS, and SRS‐SCI by diagnosis (autism/NA), with IA dimensions (Positive Insight, Focus on Child) as moderators, controlling for sex. Process for SPSS (4.2) Model 1 (Hayes 2018) was used in all analyses. Here we report the analyses that yielded significant moderation effects (detailed in Tables 6 and 7).

**TABLE 6:** Predicting SRS‐SCI by group, moderated by IA positive insight (*n* = 114).

Predictor	*b*	SE	*p*	95% CI	*F*(4, 109)	*R* ^2^
Overall model					28.73**	0.51
Group	32.12	6.75	< 0.001	[18.74–45.5]		
Positive insight	1.622	0.94	0.088	[−0.247–3.49]		
Group*positive insight	−2.68	1.13	0.020	[−4.92 to −0.43]		
Sex (Cov)	4.871	1.967	0.015	[0.97–8.77]		

Abbreviations: IA, insightfulness assessment; SRS–SCI, Social Responsiveness Scale–Social Communication Index.

** *p* < 0.001.

**TABLE 7:** Predicting SSIS‐SS by Group, moderated by IA‐positive insight (*n* = 114).

Predictor	*b*	SE	*p*	95% CI	*F*(4, 109)	*R* ^2^
Overall model					6.63**	0.20
Group	−30.04	8.85	0.001	[−47.58 to −12.50]		
Positive insight	−2.06	1.24	0.088	[−4.51–0.39]		
Group*positive insight	3.73	1.49	0.014	[0.78–6.67]		
Sex (Cov)	−6.62	2.58	0.012	[−11.73 to −1.51]		

Abbreviations: IA, insightfulness assessment; SSIS‐SS, social skills improvement scales—social skills.

** *p* < 0.001.

**FIGURE 1:**
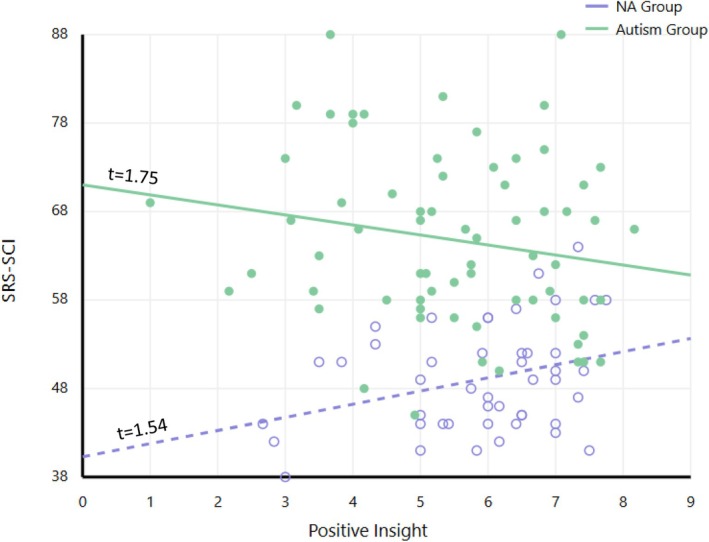
Group differences on the Social Responsiveness Scale–Social Communication and Interaction (SRS–SCI) as a function of Insightfulness Assessment–Positive Insight (IA–PoI) levels. Individual data points represent participants in the NA group (white circles) and Autism group (solid circles). The figure reveals a significant moderation effect with divergent patterns: The NA group shows a non‐significant positive trend while the Autism group displays a negative trend. Therefore, as IA‐POI increases, the difference in social communication scores between Autism and NA groups decreases. Note that higher SRS‐SCI scores indicate poorer social competence.

The moderation analysis for SRS‐SCI indicated a significant direct effect of group, as well as a moderation effect for Positive Insight (*b* = −2.68, SE = 1.134, *t* = −2.36, *p* = 0.03). Johnson–Neyman analysis revealed that as Positive Insight levels increased, the difference in SRS‐SCI scores between the Autism and NA groups decreased (Table 6, Figure 1).

**FIGURE 2:**
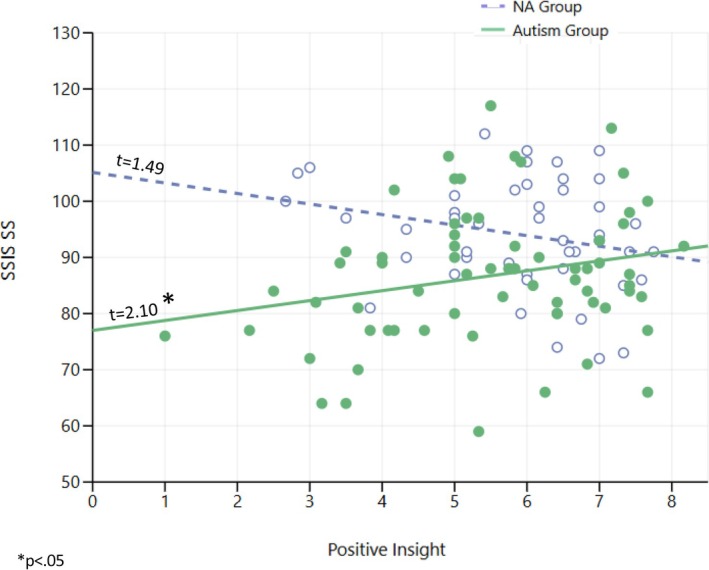
Group differences on the Social Skills Improvement System (SSIS) as a function of Insightfulness Assessment–Positive Insight (IA–PoI) levels. Individual data points represent participants in the NA group (white circles) and Autism group (solid circles). The figure illustrates a significant moderation effect with contrasting patterns between groups: The NA group shows a non‐significant negative trend while the Autism group demonstrates a significant positive association. Thus, as IA‐PoI increases, the gap in social competence between Autism and NA groups decreases. Note that higher SSIS‐SS scores represent better social competence.

In addition, as predicted, the results of the SSIS‐SS analysis revealed a significant direct effect for group, as well as a moderation effect for Positive Insight (*b* = 3.728, SE = 1.486, *t* = 2.508, *p* = 0.014). Johnson–Neyman analysis indicated that as PI levels increased, the gaps in SSIS‐SS between the Autism and NA groups decreased (Table 7, Figure 2).

To examine the moderating role of self‐reported PI on the association between having autism and the child's social competence, nine moderation analyses were conducted, predicting VABS Socialization, SSIS‐SS, and SRS‐SCI by diagnosis (Autism/NA), with PRFQ scale scores (Pre‐mentalization, Certainty in Mental States, Curiosity in the child's mental states) as moderators, controlling for sex. Process for SPSS (4.2) Model 1 (Hayes 2018) was used in all analyses. Here we report the results of the analysis that has yielded a significant moderation effect.

**TABLE 8:** Predicting VABS socialization by group, moderated by PRFQ‐PM (*n* = 114).

Predictor	*b*	SE	*p*	95% CI	*F*(4, 109)	*R* ^2^
Overall model					23.01**	0.46
Group	−2.69	4.09	0.51	[−10.80–5.42]		
PRFQ‐PM	1.44	1.72	0.40	[−1.96–4.84]		
Group* PRFQ–PM	−5.90	2.14	0.01	[−10.15 to −1.65]		
Sex (Cov)	0.27	1.85	0.88	[−3.40–3.94]		

Abbreviations: PRFQ–PM, Parent Reflective Functioning Questionnaire–Pre‐Mentalization; VABS, Vineland Adaptive Behavior Scales.

** *p* < 0.001.

**FIGURE 3:**
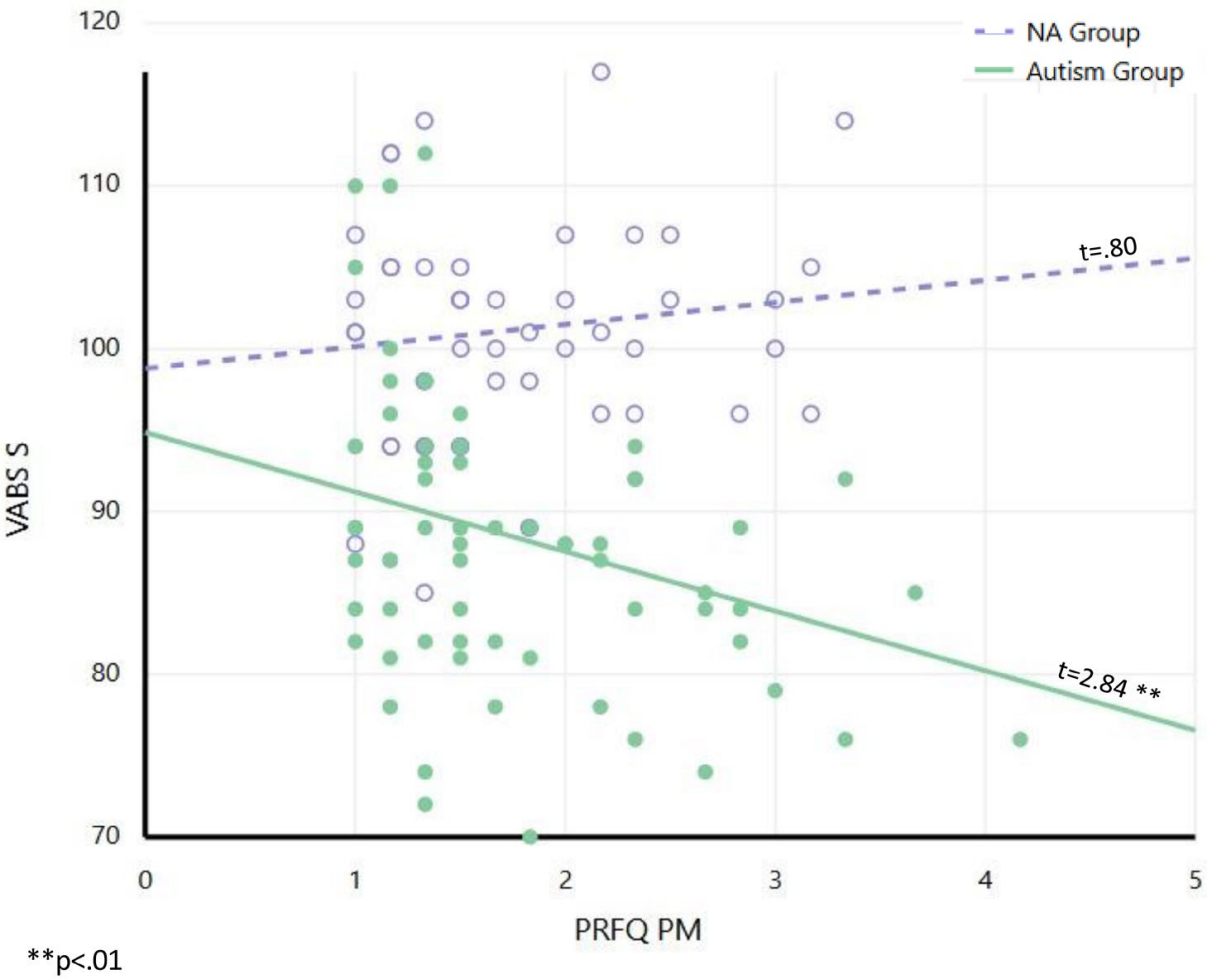
Group differences on the Vineland Adaptive Behavior Scales–Socialization Domain as a function of Parental Reflective Functioning Questionnaire–Pre‐Mentalizing (PRFQ–PM) scores. Individual data points represent participants in the NA group (white circles) and Autism group (solid circles). The figure demonstrates a significant moderation effect with a marked difference in slopes between groups: The NA group shows a non‐significant positive trend while the Autism group displays a significant negative association. Hence, as PRFQ‐PM scores increase, the gap in VABS socialization between the Autism and NA groups widens. Note that higher PRFQ–PM scores indicate poorer parental reflective functioning, and lower VABS Socialization scores represent poorer social competence.

As predicted, the results of the VABS Socialization analysis revealed a significant moderation effect for PRFQ‐PM (*b* = −2.62, SE = 2.20, *t* = −2.75, *p* < 0.01). Johnson–Neyman analysis indicated that as PRFQ‐PM levels increased (indicating poorer PI) the VABS Socialization gaps between the Autism and NA groups widened (Table 8, Figure 3).

## 4 Discussion

The current study aimed to examine the differences in parental insightfulness (PI) of parents to autistic and NA children and its relation to children's social competence. Our findings provide novel insights into PI in the context of peer interactions. To the best of our knowledge, this is the first study to investigate PI in a social context, examining how parents, and specifically autistic children's parents, understand their child's mind while the child interacts with a peer. This context was chosen due to the key role autistic children's parents play in shaping their children's social competence (Park et al. 2023). To represent PI more holistically, following previous studies (e.g., Henrikson 2023), we have also used a self‐report questionnaire to examine parents' insightfulness regarding *their own* relationships with their child, which is the more commonly used context when studying PI. Our examination also highlighted different perspectives of social competence: the VABS assessed functional social skills (Sparrow et al. 2016), the SSIS focused on specific social competencies (Greenham et al. 2017), and the SRS‐SCI evaluated social communication challenges typical in autism (Constantino et al. 2003).

Our results revealed several new findings regarding PI. First, we found that measuring PI using the IA in a peer interaction context maintains the same factor structure found in parent–child interaction. Second, a nuanced picture was demonstrated while examining PI in different contexts: When examining PI in the context of children's social interaction with a peer, autistic children's parents maintained the same level of positive insightfulness as parents of NA children, but showed lower focus on their child, more concern, and less acceptance. However, when examining PI in their own relationship with the child, no differences were found between parents of autistic and NA children. Finally, we found a moderating effect of PI on the association between children's diagnosis and their social competence, so that better PI was associated with smaller social competence differences between autistic and NA children. Next, we elaborate the discussion on each of these findings.

### 4.1 Using the IA to Examine PI in a Social Context

Results of the factor analysis conducted on the IA scores revealed two factors: *Positive Insight*, that is, parents' ability to describe the motives that underlie their child's behavior in an open, coherent, and accepting way; and *Focus on Child*, which is the parents' capacity to view their child as separate from themselves, while focusing on the child's perspective rather than on their own concerns and needs. This two‐factor structure is consistent with previous studies of the IA concerning child–parent dyads (Gomez et al. 2018; Koren‐Karie and Oppenheim 2018). This finding supports the IA's structural validity when examining PI in a peer interaction context, providing evidence for its robustness in various aspects relevant to child‐development and specifically to autism research.

Our examination of PI through the IA compared parents of autistic and NA children both on its 9 scales and on the two aforementioned factors. Findings indicate that, compared to parents of NA children, parents of autistic children showed similar levels of positive insight alongside lower levels of acceptance of the child's difficulties, heightened levels of concern about the child, and greater difficulty maintaining focus on their child's mind as separate from the parent's. These differences may represent the centrality of the social realm in the concerns of autistic children's parents (Azad and Mandell 2016; Estes et al. 2013). Adopting the child's perspective has been shown to require comprehension and acceptance of the difficulties associated with the child's diagnosis (Di Renzo et al. 2020; Oppenheim et al. 2009). The adapted IA paradigm may have elicited concern and difficulty accepting the child's observed social challenges, since parents of autistic children may have more limited opportunities to observe unmediated social interactions involving their child and a peer. Moreover, parents' focus on their concerns (e.g., with regards to the child's social future) may divert attention from the child's current activities (Crnic and Low 2002), affecting mentalizing and the parent's ability to focus on the child's emotions and behaviors.

### 4.2 Parents' Reports on PI in a Parent–Child Context

Despite the differences described above, in the context of parent–child relationships, parents of autistic and NA children reported similar levels of PI on all three PRFQ sub‐scales: Certainty and Curiosity regarding their child's mental states and pre‐mentalizing modes. These similarities suggest that fundamental aspects of parental mentalization remain intact in autistic children's parents, regardless of the child's neurodevelopmental challenges. Previous studies (Koren‐Karie et al. 2002; Oppenheim et al. 2009) have shown maternal insightfulness was unrelated to the children's temperament in NA children or to the severity of cognitive impairment in autistic children. This lack of effect could indicate that insightfulness depends more on parental processes than on the child's difficulty level (Feniger‐Schaal et al. 2019).

Together, our findings regarding PI differences between parents and autistic and NA children suggest that it is specifically in the context of the social world—when observing their child's interaction with a peer—that PI of parents to autistic children appears to be compromised. These findings emphasize the unique emotional and cognitive demands of the social domain (from both parent and child), and highlight the importance of including social contexts both in research on PI and in interventions aimed at improving PI among parents of autistic children.

### 4.3 The Moderating Role of PI

Our results suggest that PI, in its multifaceted forms, plays a moderating role in the social competence of autistic children, as demonstrated both through scored interviews (IA for insightfulness and VABS‐Socialization for social competence) and thorough parental self‐report (PRFQ‐PM for insightfulness and SRS‐SCI and SSIS‐SS for social competence). The moderation effect suggests that higher PI reduces the differences in social competence between autistic and NA children. Moreover, it appears that PI plays a more influential role among autistic children than among their NA peers. These convergent findings are congruent with existing empirical and theoretical literature emphasizing the central role PI plays in the socio‐emotional development of young autistic children (Shahar‐Maharik et al. 2018). For example, insightful parents were found to be more attuned during play interactions with their autistic children, which in turn contributed to the child's social competence (Di Renzo et al. 2020; Zand et al. 2014). Moreover, the current study's results extend previous research by underscoring the notion that parents' mental processes, which are translated into their actions and behaviors, may have a broader impact on the autistic child's social development. The social communication and interaction difficulties characteristic of autistic children require them to rely more on social mediation and modeling by their parents in the social realm, compared to their NA peers (Di Renzo et al. 2020). Following our findings, it can be posited that the parents' capacity to understand their autistic child in a complex and accepting manner may play a meaningful role in enhancing their efficacy as social mediators.

### 4.4 Theoretical and Clinical Implications

Theoretically, our findings can be viewed through the lens of resilience research (Luthar et al. 2000), which may view PI as a protective factor that promotes social competence in autistic children. This aligns with the emphasis resilience research places on understanding factors that enhance positive adaptation rather than solely focusing on deficit reduction. Clinically, these findings underscore the necessity of parental involvement in child intervention protocols. Leveraging parents' insights and potential as social mediators could enhance the efficacy of interventions targeting social competence for autistic children. Thus, future studies should examine the effect of PI on autistic children's gains following parent‐supported social skills interventions (e.g., Laugeson et al. 2024; Park et al. 2023), as well as the role of intervention‐induced changes in PI on children's social competence gains (Rabin et al. 2021).

### 4.5 Limitations and Future Directions

This study refers only to autistic children without intellectual or language impairments. Future studies should examine our research questions with a more representative sample of the autistic spectrum. Moreover, the assessment of social competence was limited to parent report and structured interviews. The inclusion of observational measures on children's social behavior and the collection of teacher reports on children's social competence (in addition to parents') may enhance the robustness and ecological validity of our findings. Also, following difficulties recruiting female autistic participants and male parents, we were unable to address potential sex‐related differences regarding our research questions. The insightfulness of mothers and fathers on the socialization of their boys and girls may be an interesting direction for future research. Finally, sample size limitations precluded analyses of distinct PI subscales as moderators on the association between having autism and social competence. These should be examined using a larger sample in the future.

Additional directions for future studies include an examination of parental characteristics that may affect their PI, such as their stress level, their well‐being, or the level of the parents' autistic traits. Also, the extension of the IA to an examination of PI in a peer context opens new avenues for the exploration of ways in which parents' understanding of their child's mind affects the child's social development. This approach also lends itself to other studies focused on parental mediation of social interaction—which is central for parents of autistic children (Tripathi et al. 2024). Due to the continuous involvement of parents in the life of autistic children, which extends into adulthood, the paradigm proposed in the current study may be applied to assess PI regarding child social interactions across later developmental stages. For example, parent‐teen reciprocity has been found to predict autistic adolescents' social conversation skills with an unfamiliar peer (Rabin et al. 2019) but did not examine the role PI plays in this association. Future studies could involve parents observing their older child's social interaction, assessing PI in this context, and examining its relation to the child's social competence.

## 5 Conclusion

This study demonstrated the multifaceted manifestation of the insightfulness of autistic children's parents, who maintain intact positive insight while showing low focus and high concern when watching their child's peer interactions. Findings also illuminate the significant role parental insightfulness plays in the social lives of autistic children, which may at least partly mitigate the social difficulties these children encounter. Our findings highlight the importance of PI‐focused interventions for parents of autistic children.

## Conflicts of Interest

The authors declare no conflicts of interest.

## Supporting information


**Appendix S1:** Insightfulness Assessment Protocol.
**Appendix S2:** Insightfulness Assessment subscales table.
**Appendix S3:** Means (SD) of PRFQ Scales in the TD\ASD Groups.

## Data Availability

The data that support the findings of this study are available on request from the corresponding author. The data are not publicly available due to privacy or ethical restrictions.
