# Supplementary material for: Parental Insightfulness and Its Association With Social Competence in Autistic and Non‐Autistic Children
Source: Autism Res. 2025 Oct 15;18(12):2508–20. doi: 10.1002/aur.70127 (PMC12729492; doi:10.1002/aur.70127)
Supplement: Supplementary file 1 — Appendix S1: Insightfulness Assessment Protocol. Appendix S2: Insightfulness Assessment subscales table. Appendix S3: Means (SD) of PRFQ Scales in the TD\ASD Groups. [file AUR-18-2508-s001.docx]

Appendix 1 - Insightfulness Assessment Protocol

Appendix 2 - Insightfulness Assessment subscales table

Appendix 3 - Means (SD) of PRFQ Scales in the TD\ASD Groups

**Appendix 1 - Insightfulness Assessment Protocol**

Prologue:

We will now view three video segments in which (child’s name) and his friend were videotaped. Each segment is two minutes long. In the first segment ______ and his friend played ‘Simon says’. In the second segment they played a ‘Jenga’ game, trying together to prevent the brick tower from collapsing. In the third segment ______ and his friend engaged in a free drawing activity.

We, as people who try to understand children, have some ideas about what children think and feel in different situations, but today we are interested to learn from *you*, as somebody who knows _____ very well, what you think about these issues.

What I will now ask you to do is to watch the first video segment and to try to think what went through _____ mind. What was he feeling and thinking during the segment? I will stop the video after two minutes and ask you some questions. Is everything clear? O.K., we can start watching.

# The first three parts

After viewing the video segment:

1. What do you think went through ______ mind? What did he feel and think during this segment?

Clarifying Questions:

* If the parent doesn’t respond directly to the segment, rather to a different segment or to everyday life, ask again: “What do you think he felt in this specific segment?”

* If the parent describes a specific characteristic of the child, but does not explain where in the segment he saw this characteristic, ask: “Where in the segment did you see that ______is (the characteristic)?”

* If the parent describes only the feelings or the thoughts of the child (but not both), ask about the other part. For example, if the parent describes the thoughts of the child, ask: “And what do you think ____ felt in this segment?”

* After the parent provides an explanation regarding where in the segment they saw a certain characteristic, ask: “Can you give me an example from everyday life of him being (the characteristic)?” If no specific characteristic was given, ask: “Can you give me an example from everyday life of a similar situation?”

If the parent says the segment is not at all like the child’s usual behavior, ask: “Can you explain the difference between how the child is usually and how he is in this segment?”

2. Is this segment characteristic of ______ more generally? Does it tell you something about his personality or about his characteristics?

Clarifying Questions:

* If the parent answers only with a “yes” or a “no”, ask: “In what way is this segment characteristic (or not characteristic) of the ______?

* After the parent has finished their answer, ask about each characteristic they described separately: “Where did you see him being (the characteristic) in the segment?” “Can you give me an example from everyday life of him being (the characteristic)?”

* Some parents have a hard time understanding the main question (number 2). In these cases, the interviewer can give examples of characteristics from the ones the parent has already mentioned, or ask again: “Could you see any of the characteristics in this segment?”

* After the parent answered the questions, ask: “Did you see any other characteristics in this segment?”

3. I would now like to ask you about what you felt, while watching this segment. Did anything concern you, surprise you, or make you happy, in this segment?

Clarifying Questions:

* If the parent says there was nothing special in this segment, the interviewer should explain “we are not looking for anything special. Just say any thought you have about what we saw”.

* If the parent refers to only one or two of the feelings mentioned (e.g. refer to happy, but not concerned or surprised) ask about the other parts: “You talked about what made you happy, is there anything that surprised you or concerned you in this segment?”

** After viewing the second segment the interviewer can explain: “After each segment, I will ask you the same questions.”

**The fourth part:**

1. I would now like to ask you a few general questions. First, from what we talked about during this interview until now, and from what you know about ______ in general, what characterizes _____ as a person, as a child? What makes ______ ______ (e.g., “what makes Sally Sally?”). I will write down what you say and ask you to give me examples later on.

(We are looking for a list of traits or characteristics. The interviewer should prepare a paper and pen for this part, and write down exactly all the descriptions/traits the parent gives of the child.)

Then say: I will read you the characteristics you talked about, one by one, and for each one I will ask for an example from your everyday life. In this part we are trying to get a broader picture of who ______ is. You said ______ is (first trait), can you give me a specific example from everyday life?

Clarifying Questions:

* Some parents do not give a list of traits, rather they give an example after each trait they name. If the example is of a specific incident, there is no need to ask for another example.

* Some parents find it very difficult to name more than one or two traits. In order to maintain a positive atmosphere, the interviewer can say: “Let’s start with what we have, and at the end I will ask you if you can think of anything else.” After the parent gave an example of the traits he named, the interviewer can ask: “Can you think of any other characteristics?”

2. Based on how well you know your child, are there things about him that surprise you, concern you, or make you happy?

Clarifying Questions:

* If the parent gives examples from the observation, the interviewer should clarify and say: “In this question, I am asking about your feelings in your everyday life with ______.”

* If the parent talks about some of the feelings, the interviewer should ask at the end of his answer about the feelings that were not discussed. For example, if the parent talked about what makes him happy, ask: “And is there anything that concerns or disturbs you about ______?”

3. What is the thing that, in your opinion, most characterizes the relationship between your child and yourself? (After the parent answers, ask:) What are your expectations for the future regarding your relationship with your child (if the child is 2 years old or older) for next year (if the child is one year old)?

Appendix 2 - Insightfulness Assessment subscales table

| **IA scale** | **Low** | **High** |
| --- | --- | --- |
| *Insight into the Child's Motives* | Parent does not talk about possible motives for their child's behavior | Parent tries to understand the thoughts and feelings that may underlie their child's behavior; they move freely between the videotaped observations and their knowledge about their child, draw parallels between the two, and try to gain deeper understanding. |
| *Openness/ Flexibility* | Parent is not open to the information of the videotaped observations, but rather speak about their fixed and present ideas about the child; the observation may be dismissed as not typical of the child. | Parent is open to the information arising from the videotaped observations; they compare what they know about their child with the video observations and modify their perceptions if needed. |
| *Complexity in description of child* | Parent describes the child in a uni-dimensional, one-sided way, emphasizing either only positive or only negative aspects of the child. | Parent provides a believable description of the child in which the child is described as a 'whole' with both positive and negative aspects. |
| *Maintenance of focus on child* | The child is not the focus of discussion; rather, the focus is on the parent and their feelings and thoughts or other irrelevant issues. | The child is the focus of discussion; if parent talks about themselves, it will be when they are asked to do so or regarding their parental role |
| *Richness of description of child* | Limited responses that lack substance or full responses with mostly irrelevant details. | Parent response to the interview questions in a full, comprehensive and vivid way. |
| *Acceptance/ Warmth* | Parent expresses dissatisfaction or disappointment in the child or talks about the child in a derogatory way. | Parent accepts the full range of their child's behaviors and shows tolerance and understanding towards challenging aspects; they are open about difficulties in their child's behavior and convey a deep acceptance of the child. |
|  |  |  |
| *Hostility/Anger* | Parent’s speech does not include current anger even though they can talk about behaviors that caused them to feel angry in the past. | Current anger towards the child is central feature of parent's talk; the child is described as having irritating traits, and many of his behaviors in the videotaped observations elicit anger on parent. |
| *Concern* | Parent express believe in themself and their child's capacity to cope with challenges | Parent's worry regarding the child, their parental behavior, or their relationship is a central, repetitive theme throuout the interview. |
| *Separateness from child* | Parent finds it difficult to talk about the child with a sense of clear boundaries; they may talk about the child's thoughts as if spoken out loud or refer to ideas regarding what the child might think or feel as facts. | Parent sees the child as a separate person and accepts that the child may sometimes have needs and wishes that are different or even contradictory to hers. |
| *Coherence of thoughts* | Parent speech does not convey a consistent and clear picture, and it is difficult to understand what they mean. | Parent is focused on the videotaped segments, and in their answer; they develop ideas in a consistent, connected, and relaxed way. |

**Appendix 3 - Means (SD) of PRFQ Scales in the NA/ASD Groups**

| **PRFQ scales** | **NA** | **ASD** | ***F*(1,111)** | **η^2^** |
| --- | --- | --- | --- | --- |
| PM | 1.79 (.66) | 1.76 (.72) | .00 | .00 |
| CMS | 3.81 (1.19) | 3.9 (1.01) | .05 | .00 |
| IC | 6.15 (.81) | 5.92 (.77) | 2.39 | .02 |

Note: PRFQ – Parent Reflective Functioning Questionnaire; PM - Pre-Mentalization; CMS - Certainty in Child’s Mental States; IC - Interest and Curiosity in Child’s Mental States.
